# Effects of Real-Time Pressure Map Feedback on Confidence in Pressure Management in Wheelchair Users With Spinal Cord Injury: Pilot Intervention Study

**DOI:** 10.2196/49813

**Published:** 2023-10-12

**Authors:** Tamara L Vos-Draper, Melissa M B Morrow, John E Ferguson, Virgil G Mathiowetz

**Affiliations:** 1 Center for Allied Health Professions Program in Occupational Therapy University of Minnesota Minneapolis, MN United States; 2 Division of Rehabilitation Science Department of Rehabilitation Medicine University of Minnesota Minneapolis, MN United States; 3 School of Health Professions Center for Health Promotion, Performance, and Rehabilitation Research University of Texas Medical Branch Galveston, TX United States; 4 Division of Physical Medicine & Rehabilitation Department of Rehabilitation Medicine University of Minnesota Minneapolis, MN United States; 5 Minneapolis Veterans Affairs Health Care System Minneapolis, MN United States

**Keywords:** spinal cord injury, wheelchair, pressure injury prevention, self-efficacy, pressure mapping, pressure, mapping, map, interface, spine, spinal cord, feedback, real time, mobile phone

## Abstract

**Background:**

Wheelchair users with a spinal cord injury (SCI) are at a high risk for developing pressure injuries (PIs). Performing weight shifts is a primary method of pressure management for PI prevention; however, individuals with SCI may lack confidence in their abilities to perform adequate pressure relief due to their lack of sensation. Real-time seat interface pressure mapping feedback may provide partial substitution for sensory feedback such that an individual’s confidence is improved.

**Objective:**

We aim to examine how confidence for pressure management by wheelchair users with SCI was impacted by providing access to real-time, on-demand seat interface pressure mapping feedback.

**Methods:**

Adults with SCI (N=23) completed self-efficacy questions addressing confidence around 4 factors related to performing weight shifts in this longitudinal, repeated-measures study. We evaluated the impact of providing standard PI prevention education and access to live pressure map feedback on confidence levels for performing weight shifts.

**Results:**

Access to live pressure map feedback while learning how to perform weight shifts resulted in significantly higher confidence about moving far enough to relieve pressure at high-risk areas. Confidence for adhering to the recommended weight shift frequency and duration was not significantly impacted by in-clinic education or use of pressure map feedback. Confidence that performing weight shifts reduces PI risk increased most following education, with slight additional increase when pressure map feedback was added.

**Conclusions:**

Access to live pressure mapping feedback improves confidence about performing weight shifts that relieve pressure when provided in the clinical setting and demonstrates potential for the same in the home. This preliminary exploration of a smartphone-based pressure mapping intervention highlights the value of access to continuous pressure mapping feedback to improve awareness and confidence for managing pressure.

**Trial Registration:**

ClinicalTrials.gov NCT03987243; https://clinicaltrials.gov/study/NCT03987243

## Introduction

Wheelchair users with a spinal cord injury (SCI) are at a high risk for developing pressure injuries (PIs) [1]. Heightened risk is due to motor and sensory impairments that require prolonged periods of sitting coupled with difficulty sensing pressure on the skin. PI risk for those with SCI is persistent across the life span and significantly impacts quality of life and occupational engagement when present because healing requires bedrest and time away from routine activities [2]. Individuals with SCI must learn effective self-management strategies to mitigate their risk for developing PIs [3].

During initial rehabilitation, patient education for PI prevention emphasizes techniques to redistribute pressure away from bony areas of the pelvis, where most PI occur in the SCI population [4,5]. Therapists teach new wheelchair users how to perform effective weight shifts using written materials and demonstration of techniques. Further, therapists use seat interface pressure mapping (IPM) as an effective way to visualize how pressure is distributed and to guide wheelchair positioning [6,7]. However, evidence suggests that prevention knowledge and pressure management behaviors gained during inpatient rehabilitation decay over time [8], and wheelchair users with SCI complete far fewer weight shifts than recommended and that movements are inconsistent and sporadic from day to day [9,10].

**Figure 1:**
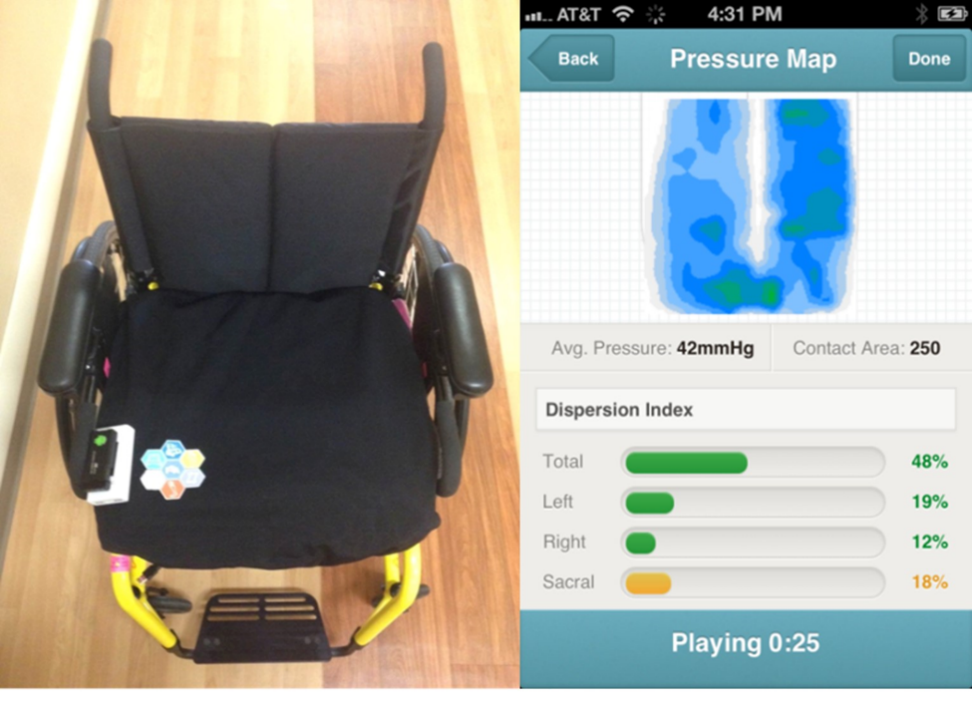
(Left) BodiTrac pressure map and wireless mPMAP hardware on top of seat cushion, (right) web-based mobile app (mPMAP). mPMAP: mobile pressure mapping app.

We posit that 1 factor to target for improving pressure management behaviors is a person’s own confidence in their ability to perform effective weight shifts [11]. Further, we hypothesize that a key aspect for improving confidence in pressure management behavior is the access to feedback about seating pressures while in a wheelchair. The natural sensory feedback is missing in individuals with SCI, so they require an alternative feedback system that can improve confidence and lead to action taken on proper pressure management. Thus, we are interested in developing and testing interventions that can improve confidence. In response to this, we have developed a mobile pressure mapping app (mPMAP) [12-14] that provides real-time pressure map display on a smartphone screen via wireless connection to the commercially available 4-way stretch BodiTrac pressure mat (Vista Medical, Inc; Figure 1).

The purpose of this study was to assess how confidence scores related to pressure management change when individuals with SCI receive (1) pressure management education alone (in-clinic), (2) education with on-demand IPM feedback (in-clinic), and (3) home use of on-demand IPM feedback (for a trial period). We tested this by surveying user’s confidence about 3 specific aspects of managing pressure and the strength of their belief that weight shifts can prevent PI. We hypothesized that use of on-demand seat IPM system would result in increased confidence across pressure management factors (PI prevention, weight shift effectiveness, weight shift frequency, and weight shift duration).

## Methods

### Ethical Considerations

Manual and power wheelchair users with complete SCI who were able to perform weight shifts or use power tilt independently participated in this study. Participants were recruited through convenience sampling from an SCI outpatient rehabilitation program and wheelchair seating clinic at a large Midwestern medical system, after the institutional review board’s approval (16-007531). Participants provided informed consent prior to data collection, and all data reported are deidentified. Participants were compensated with US $100 for completing this study. Data collection occurred between October 2016 and August 2017. Inclusion criteria required participants to use a wheelchair for a minimum of 6 hours per day, independence in performing weight shifts by leaning or by using power seat functions, and ability to independently use a smartphone. Exclusion criteria prohibited participation if there was an active PI on the pelvic region.

### Study Design

This longitudinal, within-subject, repeated measures design was conducted over a 1-month period. Participants participated in an in-clinic study visit followed by in-home data collection for 1 month.

### Interventions

We provided standard education for PI prevention that focused on weight shifts to redistribute pressure. We used videos produced by the Rehabilitation Research and Training Center on SCI that depict individuals with SCI performing the tasks [15] and printed patient education materials with drawings depicting the weight shifts [16,17]. We used IPM to provide visual feedback during the in-clinic visit (Vista Medical, Inc) and a mobile app version (mPMAP; Figure 1) for participant access to visual feedback during the in-home phase.

### Outcome Measure: Self-Efficacy (SE) Scale to Assess Confidence

We measured level of confidence for performing weight shifts using a 4-item self-efficacy (SE) survey developed for this study using the principles outlined in the “Guide for constructing self-efficacy scales” [18]. Content validity was confirmed through expert clinician review by occupational therapy and physical therapy staff on an SCI rehabilitation team. The SE questions were each rated from 1 (lowest confidence) to 100 (highest confidence) by the participants. The first SE question (Q1) targeted an individual’s outcome belief that completing weight shifts prevents PIs. The remaining questions assessed judgment of their current capability to complete weight shift maneuvers based on 3 criteria: (Q2) effectiveness (moving far enough to improve pressure distribution), (Q3) consistency (completing weight shifts every half hour), and (Q4) duration (holding weight shifts for 2 minute).

### In-Clinic Visit

A preintervention baseline SE measure was obtained with the 4-item SE survey. Next, to ensure a consistent level of education about how to redistribute pressure through leaning or use of power tilt, we provided structured education for performing weight shift maneuvers for PI prevention. Participants practiced completing the weight shift maneuvers with feedback from this study team’s seating and mobility expert. For full forward and side leans, participants were asked to move as far as possible in each direction and for partial forward and side leans, they leaned far enough to rest elbows on lap or on armrests or tires, similar to the approach used in earlier studies [19]. *The structured weight shift maneuver protocol was completed as follows:* (1) full forward lean, (2) full right-side lean, (3) full left-side lean, (4) partial forward lean, (5) partial right-side lean, and (6) partial left-side lean. Weight shift maneuvers were determined completed for full leans when the participant moved safely as far as they could in the intended direction which included holding on to foot plates for forward lean or the tire for the side leans. For the partial lean, participants were instructed to lean half as far as their full lean. The lean approach described was used because each individual had variable levels of control and ability to lean; hence the maneuvers and pressure offload goals were customized for each user. For power tilt users, full weight shift required tilting back as far as the chair allowed (45-55 degrees) and to 30 degrees for partial tilt [20]. After providing education, a second administration of the SE items was completed.

Next, we introduced use of IPM feedback using a clinical system with computer display visible to the participants. Real-time pressure distribution feedback was shown to the participants as they completed a series of weight shift movements. Participants were instructed to observe the changes in pressure distribution on the screen while they practiced weight shifts. After exposure to IPM feedback, participants answered the SE survey a third time.

### In-Home Use of mPMAP

At the conclusion of the in-clinic visit, each participant was provided with an iPhone with a 30-day prepaid data plan and an mPMAP system to use at home. Participants were instructed that the testing period was 30 days and that they would alternate across weeks in which they would or would not use the system (an ABAB design). This study’s period began with a 1-week period of using the system, followed by 1 week not using the system, followed by a second and final week using the system, followed by a final week of this study not using the system. All participants demonstrated an ability to access and use mPMAP independently through teach-back observation. Daily activity logs, with assigned days for accessing the mPMAP feedback highlighted, were provided to each participant to record days of mPMAP use and comments on usability of the system. Participants were contacted within 2 days of starting the home use period to repeat the SE survey for reliability testing of the items and then again during each of the alternating periods of mPMAP use and without mPMAP use during the in-home data collection period. In total, participants completed the SE survey 5 times during the at home period.

### Data Analysis

Statistical analyses were carried out using SPSS statistical software (version 24.0; IBM Corp) for Windows [21]. Because the data were skewed, Wilcoxon signed rank test was the most appropriate statistical test [22]. We calculated effect size (*r*) with the recommended method for nonparametric repeated measures, 
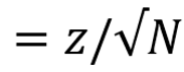
, and interpreted the effect size of *r* using Cohen guidelines: large effect size=0.5, medium=0.3, and small effect size=0.1 [23]. We made 3 within-person, pairwise planned comparisons for each SE item confidence score: (1) baseline measure versus posteducation, (2) posteducation versus posteducation IPM feedback, and (3) with mPMAP use at home versus without use of mPMAP at home. Because these were planned comparisons, we did not apply an adjustment for multiple comparisons.

## Results

### Participant Characteristics

There were no statistically significant characteristic differences between those who completed the in-clinic (N=23) and in-home (N=16) data collection periods (Table 1). The sample was heterogeneous with representation across injury level, wheelchair and cushion types, PI experience, time since injury, and age. The sex distribution at the in-clinic visit was 78.3% (n=18) male and 21.7% (n=5) female, and in the home phase, the distribution shifted to 68.8% (n=11) male and 31.3% (n=5) female as 7 men did not complete the in-home data collection.

**Table 1:** Participant characteristics.

Variables		In-clinic visit (n=23)	In-home phase (n=16)
**Gender, n (%)**			
	Male	18 (78.3)	11 (68.8)
	Female	5 (21.7)	5 (31.3)
**SCI^a^ level, n (%)**			
	Cervical	10 (43.5)	6 (37.5)
	Thoracic	12 (52.2)	9 (56.3)
	Lumbar	1 (4.3)	1 (6.3)
**Wheelchair, n (%)**			
	Manual	14 (60.9)	10 (62.5)
	Power with tilt	8 (34.8)	5 (31.3)
	Power without tilt	1 (4.3)	1 (6.3)
**Seat cushion, n (%)**			
	Offloading, noncustom	6 (26.1)	5 (31.3)
	Immersion	16 (69.6)	11 (68.8)
	Alternating air (powered)	1 (6.3)	0 (0)
**Pressure injury history, n (%)**			
	Pelvic pressure injury	11 (47.8)	9 (56.3)
	Surgical repair	10 (43.5)	7 (43.8)
**Onset time (years), n (%)**			
	0-5	7 (30.4)	3 (18.8)
	6-15	4 (17.4)	3 (18.8)
	16 or older	12 (52.2)	10 (62.5)
Age (years), mean (SD), median (IQR)		42.17 (13.16), 39 (21-65)	42.5 (12.38), 40 (27-63)
Years since onset, mean (SD), median (IQR)		15.74 (11.77), 18 (1-43)	18.13 (11.40), 20 (2-43)

^a^SCI: spinal cord injury.

### PI Prevention

Confidence that performing weight shifts prevents PI increased significantly from baseline (mean 85.2, SD 23.7) to after standard education was provided (mean 90.2, SD 14.2; *P*=.02), with a large effect size (*r*=–0.503). Score increased further (mean 94.3, SD 9.9) after introduction of IPM feedback in clinic and remained above mean score of 93.8 (SD 9.9) for the 1-month at-home phase of this study (Tables 2 and 3), but this increase was not statistically significant, and the effect size was small.

**Table 2:** Mean self-efficacy scores across time for 4-items in response to: “I believe I am able to…”

	Prevent pressure injury using weight shifts		Move far enough to relieve pressure	
Time	Mean (SD)	95% CI	Mean (SD)	95% CI
Baseline^a^	85.2 (23.7)	75.0-95.5	79.8 (25.8)	68.6-90.9
Posteducation^a^	90.2 (14.2)	84.1-96.3	85.7 (17.7)	78.0-93.3
Education + map^a^	94.3 (9.9)	90.1-98.6	97.0 (5.6)	94.5-99.4
Test-retest^b^	94.5 (10.1)	89.6-99.4	97.6 (4.2)	95.6-99.7
mPMAP^c^ 1^d^	93.8 (9.9)	88.7-98.9	95.3 (8.9)	90.7-99.9
No mPMAP 1^e^	93.8 (9.4)	88.7-98.8	91.9 (11.8)	85.6-98.2
mPMAP 2^f^	96.2 (7.7)	91.5-100.8	97.3 (6.0)	93.7-100.9
No mPMAP 2^g^	95.9 (7.4)	91.0-100.9	95.0 (8.7)	89.2-100.8

^a^n=23.

^b^n=19.

^c^mPMAP: mobile pressure mapping app.

^d^n=17.

^e^n=16.

^f^n=13.

^g^n=11.

**Table 3:** Mean self-efficacy scores across time for 4-items in response to: “I believe I am able to…”

	Perform weight shifts every 30 minutes		Hold weight shifts for duration of 2 minutes	
Time	Mean (SD)	95% CI	Mean (SD)	95% CI
Baseline^a^	82.3 (28.8)	69.9-94.8	88.9 (25.0)	78.1-99.7
Posteducation^a^	89.8 (20.1)	81.1-98.5	92.4 (16.5)	85.3-99.5
Education + map^a^	91.5 (16.9)	84.2-98.8	92.0 (16.7)	84.7-99.2
Test-retest^b^	94.7 (10.2)	89.8-99.7	94.2 (10.2)	89.3-99.1
mPMAP^c^ 1^d^	95.0 (10.5)	89.6-100.4	95.9 (9.2)	91.1-100.6
No mPMAP 1^e^	89.7 (15.6)	81.3-98.0	93.4 (9.8)	88.2-98.7
mPMAP 2^f^	95.4 (9.7)	89.5-101.2	92.3 (13.6)	84.1-100.5
No mPMAP 2^g^	91.8 (12.5)	83.4-100.2	91.4 (14.2)	81.9-100.9

^a^n=23.

^b^n=19.

^c^mPMAP: mobile pressure mapping app.

^d^n=17.

^e^n=16.

^f^n=13.

^g^n=11.

### Weight Shift Effectiveness

Confidence for knowing one has moved far enough to effectively redistribute pressure during a weight shift had the lowest mean score out of the 4 questions at baseline (mean 79.8, SD 25.8) with slight increase after standard education was delivered (mean 85.7, SD 17.7; Tables 2 and 3). However, after given access to IPM feedback, the mean confidence score increased significantly (mean 97.0, SD 5.6; *P*=.002), with a large effect size (*r*=–0.642; Table 4). This was the largest effect size observed across questions and comparisons. Additionally, during at-home IPM access, the mean confidence score was significantly higher (mean 95.3, SD 8.9) than period of time without IPM access (mean 91.9, SD 11.8), *P*=.02, again, with a large effect size (*r*=–0.566; Table 4).

**Table 4:** Wilcoxon signed rank tests for self-efficacy scores.

I believe I am able to:	Baseline versus posteducation (N=23)			Posteducation versus education + IPM^a^ feedback (N=23)			mPMAP^b^ use versus no mPMAP use at home^c^ (N=16)		
	*Z* ^d^	*P* value	*r* ^e^	*Z*	*P* value	*r*	*Z*	*P* value	*r*
Prevent pressure injuries by performing weight shifts at regular intervals when I am in my wheelchair.	2.41	.02^f^	0.503	1.414	.16	0.295	0.213	.83	0.053
Move far enough during weight shifts to relieve pressure at my high-risk areas.	0.088	.38	0.018	3.077	.002^f^	0.642	2.264	.02^f^	0.566
Consistently perform weight shifts at least every half hour during the day.	1.826	.07	0.381	0.736	.46	0.153	1.59	.11	0.398
Hold my weight shifts for two full minutes as recommended for at least half of my weight shifts.	0	>.99	0	0.378	.71	0.079	1.361	.17	0.34

^a^IPM: interface pressure map.

^b^mPMAP: mobile pressure mapping app.

^c^The first week of mPMAP use was compared with first week of non-mPMAP use at home.

^d^*Z*: Wilcoxon signed rank test statistic.

^e^*r*: effect size (Z/√N; Cohen).

^f^*P*<.05.

### Weight Shift Frequency and Duration

Confidence for performing weight shifts at the recommended frequency of every 30 minutes and holding for duration of 2 minutes did not change significantly from baseline measure to following standard education, between standard education and access to IPM feedback, or with access to IPM feedback at home (Tables 2-4).

## Discussion

### Primary Findings

These results provide evidence that access to IPM feedback improves confidence around pressure management by wheelchair users with SCI, and specifically around awareness of how to move to redistribute pressure effectively. Each of the 4 questions (PI prevention, weight shift effectiveness, weight shift frequency, and weight shift duration) were grounded in SE theory and each addressed a specific aspect of pressure management.

### PI Prevention

The first question focused on the outcome expectation that one is able to prevent PIs by performing weight shifts. We predicted that IPM feedback would increase confidence more than standard education; however, the most significant increase occurred immediately after we provided standard patient education. Confidence remained higher than baseline after IPM was introduced and while IPM was used at home. Further, because this study had just a 1-month in-home period, we do not yet know if ongoing access to IPM would reduce the knowledge decay observed in other studies [8] that occurs in the first year after education is provided to those newly injured. Other studies have provided evidence that education provided to individuals with SCI about PI prevention improves SE or knowledge, but they have not specifically addressed confidence around performance of weight shifts as we have demonstrated in this study.

### Weight Shift Effectiveness

We observed the strongest impact of IPM feedback on the second survey item which queries confidence in knowing how far to move to effectively redistribute pressure. Because lack of sensation is a major PI risk factor in the SCI population, we could expect that awareness of pressure would improve with a surrogate visual feedback mechanism provided by sensors that measure pressure directly between the person and their seat cushion. By increasing awareness of pressure using IPM, the participants in our study reported significantly improved confidence about their ability to manage pressure through movement. Confidence decreased when the IPM feedback was removed during the in-home phase of this study, signaling that perhaps access to IPM feedback may need to be continuous or on-demand as a long-term compensatory strategy. While seat IPM has been criticized for limited effectiveness in predicting those at risk when used as an assessment from 1 clinical assessment [24], it does not negate the potential value of IPM for prevention when used as real-time feedback provided directly to the end user [25].

### Weight Shift Frequency and Duration

In total, 2 of the SE questions targeted the timing of weight shifts, and they appeared unaffected by introduction of IPM feedback which may be due to the lack of reminders or alarms in the system. Wheelchair users with SCI have been shown to not move as frequently as guidelines suggest, which could explain the lower confidence scores around these 2 specific items. If the questions were worded differently, to suggest confidence in adhering to their personal goals for frequency and duration of performing weight shifts, the response may have been different. Since concluding data collection in this study which used the initial prototype on-demand pressure mapping system, we have made new developments that include features desired by veterans who have SCI [26]. The updated system includes user-controlled settings for reminders and alerts which may prove to be more effective for improving confidence for these aspects of weight shift performance.

### Future Research

Future research should explore the impact of IPM combined with reminders to perform weight shifts and alerts to high pressure on weight shift confidence and also subsequent impact on pressure management including weight shift behaviors when using the compensatory strategies. The simple 4-item scale used in this study that specifically addresses weight shift performance factors could be useful in clinical practice to determine where the wheelchair user feels least confident and then interventions could focus on that specific aspect of weight shifts when discussing self-management strategies. Additionally, the current method of placing a pressure mat on top of a wheelchair cushion has known negative effects including sliding, challenges with postural stability, and moisture-wicking; hence, future research will explore alternative methods to capture pressure data with sensors that can overcome the issues related to placing a mat in the interface between the user and the cushion.

### Limitations

Due to lack of access to participant level app interaction, we do not know how often the participants accessed the pressure map feedback in the home. Further, we did not incorporate self-reported use of the system into our analysis. The weight shift protocol performed used a qualitative approach to guide participants. Our sample size was less than 25, and heterogeneous which reduced our ability to consider covariates such as level of injury or prior experience with PI into the results. The results of this study serve to test whether visual on-demand pressure map feedback increases confidence toward pressure management; however, the results do not provide evidence toward the translation of high confidence into increased adherence to improved pressure management strategies.

### Conclusions

Our results provide evidence that on-demand pressure map feedback, when used to guide weight shifts, has a positive impact on wheelchair user’s confidence in performing effective weight shifts to reduce pressure. Additional exploration could consider how confidence levels respond to technologies that more specifically target weight shift timing. Clinical efficacy studies are recommended to explore how these technologies impact PI incidence over time.

## Abbreviations

IPM: interface pressure mapping
mPMAP: mobile pressure mapping app
PI: pressure injury
SCI: spinal cord injury
SE: self-efficacy

## Data Availability

The data sets generated or analyzed during this study are available from the corresponding author on reasonable request.
